# Characteristics of rifampicin-resistant tuberculosis detection in China, 2015–2019

**DOI:** 10.1186/s40249-021-00883-8

**Published:** 2021-07-17

**Authors:** Wei Su, Yun-zhou Ruan, Tao Li, Xin Du, Jia-wen Jiang, Ren-zhong Li

**Affiliations:** National Center for Tuberculosis Control and Prevention, China CDC, No.155 Changbai Road, Changping District, Beijing, 102206 People’s Republic of China

**Keywords:** Rifampicin-resistant tuberculosis, Case detection, Detection policy, China

## Abstract

**Background:**

The very high burden of rifampicin resistance tuberculosis (RR-TB) and the very low detection of RR-TB cases are a major challenge that China has been facing. This study analyzed the characteristics of RR-TB detection in China after the change of RR-TB detection strategy since 2015, aiming to provide reference and evidence for the development of more precise national drug resistance tuberculosis prevention and control policy.

**Methods:**

We extracted data related to rifampicin resistance screening from the national Tuberculosis Information Management System (TBIMS) from 2015 to 2019, and used descriptive research methods to analyze the screening rate of presumptive RR-TB, the number and duration of RR-TB patients detected and drug resistance testing methods in each year. Chi-square test was used to compare the differences in component ratio or rate between years, and Kruskal Wallis test was used to compare the differences in median days for detection of RR-TB patients in each year.

**Results:**

A total of 68,200 RR-TB cases were detected during 2015–2019, of which 48.1% were new cases. The number and detection rate of RR-TB cases increased year by year, from 10 019 and 14.3% in 2015 to 18 623 and 28.7% in 2019, respectively. Of the bacteriologically confirmed TB cases, 81.9% were tested for RR in 2019, a considerable increase from 29.5% in 2015. In 2019, only 41.0% of RR-TB cases had fluoroquinolones (FQs) susceptibility testing performed, and this proportion has been declining year by year since 2016. The proportion of application of rapid molecular tools increased from 24.0% in 2015 to 67.1% in 2019, and the median days to obtain RR results was significantly shortened. In 2019, 76.0% of RR-TB cases were diagnosed as presumptive RR-TB in county-level hospitals.

**Conclusions:**

After China modified the RR-TB detection strategy, the screening rate of RR and the number of RR-TB cases increased significantly. The RR testing methods now predominantly utilize rapid molecular tools. However, comprehensive measures should be implemented to close the gap in the detection of RR-TB cases. It is imperative to take FQs susceptibility testing seriously and effectively strengthen the laboratory capacity of county-level hospitals.

**Graphical Abstract:**

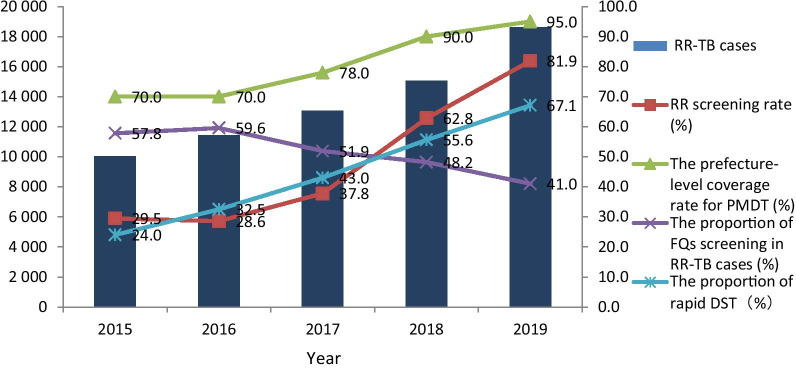

## Background

Drug-resistant tuberculosis (TB) remains an important public health concern worldwide, especially rifampicin resistance TB (RR-TB). RR-TB is defined as any resistance to rifampicin, including mono-resistance, multidrug resistance, polydrug resistance [1]. In 2019, there were 465 000 RR-TB cases worldwide; 56% were undetected. Even when the diagnosis was made, only 57% achieved a successful treatment outcome [2].

China is a country with a high burden of RR-TB. The number of cases in China accounts for 14% of global RR-TB cases, ranking second after India. The rifampicin resistance (RR) rates of new and retreated TB cases were 7.1% and 23.0%, respectively, which were higher than the global levels of 3.3% and 17.7% [2]. China initiated the Programmatic Management of Drug-resistant Tuberculosis (PMDT) in 2006 with the support of the Global Fund Project. By the end of June 2014, when the Global Fund Project closed in China, nearly one-third of the prefectures in 30 provinces (China has 31 provinces) had implemented PMDT [3, 4]. However, due to limited resources and personnel capabilities, traditional phenotypic drug susceptibility testing (DST) was mainly used at this stage and only the drug susceptibility of RR high-risk groups was tested. Not only did it take longer to diagnose patients, but fewer RR-TB patients were detected [5]. In 2012–2014, China only detected 5.0%, 7.7%, and 11.3%, respectively, of RR-TB cases estimated by the World Health Organization (WHO) [6–8]. The very high burden of RR-TB and the very low RR-TB case detection had been the major challenge facing China since the implementation of PMDT in 2006.

WHO’s End TB Strategy calls for the early diagnosis of TB and for universal drug-susceptibility testing [9]. In order to achieve the goal of End TB Strategy and solve the issue of lower detection of RR-TB cases, when the Global Fund Project ended and since 2015 China has modified the RR-TB detection strategy to scale up drug resistance screening from only high-risk groups of RR-TB to all bacteriologically confirmed TB cases. In particular, the “13th Five-Year National Tuberculosis Prevention and Control Program (2016–2020)” (13th Five-Year TB Program) adopted the expansion strategy of RR-TB detection and called for the priority application of rapid molecular technology for drug susceptibility testing to shorten the diagnosis time. It is required that the screening rate of high-risk groups for RR reach 95% and achieve full coverage of PMDT by the end of the 13th Five-Year Plan in 2020 [10]. In order to fulfill the commitment of the political declaration of the first United Nations (UN) High-Level Conference on Tuberculosis held in 2018 [11], the Chinese government issued the “Stop Tuberculosis Action Plan (2019–2022)” in 2019 and set a more ambitious target for rifampicin resistance testing. The new target is to have 90% of bacteriologically confirmed TB cases screened for rifampicin resistance by 2022 [12].

Benefiting from the accumulated experience during the Global Fund Project and aggressive promotion of the national 13th Five-Year TB Program and the Stop TB Action Plan (2019–2022), the number of RR-TB cases detected in China has increased significantly every year since 2015. In order to better understand the changes of RR-TB detection in China after 2015 and provide evidence for the national drug-resistant TB prevention and control strategy, we present a review of the current status of RR-TB detection in China from 2015 to 2019 based on data of the national Tuberculosis Information Management System (TBIMS) [13]. This review focuses on the RR screening rate, the number of RR-TB cases detected, and the application of rifampicin resistance diagnostic tools as well as the change in the source of presumptive RR-TB cases. Since fluoroquinolones (FQs) are another core anti-tuberculosis drug besides rifampicin, whether there is resistance to FQs has a great impact on the choice of chemotherapy regimens and treatment outcomes [14, 15]. Moreover, WHO defines at least 80% of bacteriologically confirmed TB patients undergoing rifampicin resistance testing and at least 80% of RR-TB patients undergoing FQs resistance testing as good testing coverage [2]. Therefore, we also analyzed fluoroquinolones resistance (FQR) among RR-TB patients. This study aims to provide reference and evidence for the development of more precise national drug resistance tuberculosis prevention and control strategy in the future.

## Methods

### Data sources

The data was extracted from the national TBIMS. The bacteriologically confirmed TB cases notified in the TBIMS and of which were diagnosed with RR-TB cases were included in our analysis from January 1, 2015 to December 31, 2019. Only tuberculosis-related information in TBIMS was used, and confidential information such as patients’ personal identity was not involved.

### DST methods and procedure

China currently applies rapid molecular and traditional phenotypic diagnostic tools to test the susceptibility of anti-tuberculosis drugs. Rapid molecular assays include Xpert MTB/RIF (Xpert) and line probe assays (LPAs), which are recommended by the WHO [16], as well as domestically produced MeltPro TB assays (MeltPro) [17] and Genechip [18]. MeltPro can detect isoniazid, rifampicin, and FQ resistance. Genechip can detect isoniazid and rifampicin resistance. Bacteriologically confirmed TB cases are the subjects of DST, and at least rifampicin, isoniazid, FQ, and second-line injections are tested for drug susceptibility. If rapid molecular methods available, rapid DST will be preferred. In the implementation of DST, the identification of *Mycobacterium tuberculosis* complex and non-tuberculous mycobacteria (NTM) was carried out. The current rifampicin resistance has ruled out NTM infection.

The DST procedure is first to determine whether a presumptive TB patient is bacteriologically confirmed and then perform DST on the bacteriologically confirmed TB patient using molecular or traditional testing tools. Specifically, county-level TB designated hospitals diagnose bacteriologically confirmed TB cases, of which counties equipped with Xpert conduct rapid RR testing for bacteriologically confirmed TB cases. The confirmed RR-TB cases will be referred to the prefecture-level designated TB hospitals for DST of other drugs; those counties without Xpert need to transport sputum smear-positive specimens or culture-positive strains to the prefecture-level TB designated hospitals for DST of rifampicin and other drugs.

### Laboratory quality control

China has established a complete laboratory network system at the national, provincial, prefecture and county levels and has a sound quality assurance (QA) system [19]. Laboratories at all levels not only carry out quality control (QC) but also accept external quality assessments (EQA). All laboratories are qualified to carry out testing only after passing quality assessment. Laboratories that carry out DST need to undergo a proficiency test organized by the National Tuberculosis Reference Laboratory once a year. County-level laboratories that carry out sputum smear microscopy need to undergo blind re-examination by prefecture-level laboratories every quarter.

### Definitions

A bacteriologically confirmed TB case refers to sputum smear positive, only culture positive, or only positive molecular testing positive [20]. The presumptive RR-TB patient in this study refers to a TB patient who is bacteriologically confirmed. High-risk groups refer to at least one of the following: (a) chronic TB patients/failure of retreatment TB patients, (b) close contact with a known RR-TB patient, (c) new TB patients of initial treatment failure, (d) relapsed or returned TB patients or (e) new TB patients remaining sputum culture or smear positive at the end of the 2nd month after treatment [21].

### Statistical analysis

Relevant data was derived from TBIMS and a descriptive analysis method was used to compare the changes of RR-TB cases detection in 2015–2019. The enumeration data was described by component ratio or rate. The comparison of inter-annual differences was tested by Chi-squared test. The measurement data was presented by medians with an interquartile range (IQR), and the comparison of inter-annual differences was tested by Kruskal Wallis test. All *P* values are two-tailed; a value less than 0.05 was considered statistically significant. All statistical analyses were done with SPSS software version 20.0 (SPSS Inc., Chicago, IL, USA).

## Results

### The status of RR-TB detection and FQs resistance testing

A total of 68 200 patients were detected in 2015–2019, of which 48.1% were new patients. Only 50.3% of all RR-TB patients were tested for FQ susceptibility. The number of RR-TB patients detected, the RR screening rate, and the coverage rate of PMDT increased year by year. However, during 2015–2017, the first 3 years when the RR-TB case detection strategy changed, the rising trend was not obvious and the RR screening rate even declined slightly in 2016. Since 2018, in the late period of the 13th Five-Year TB Program, there has been a significant increase. By 2019, the number of patients detected, the RR screening rate, and the coverage rate of PMDT reached the highest level, 1.9 times (18 623/10 019), 2.8 times (81.9%/29.5%) and 1.4 times (95/70) that of 2015 respectively. The proportion of FQ susceptibility testing in RR-TB cases had been declining year by year since 2016, and was the lowest in 2019 (Table 1).

**Table 1:** The status of RR-TB detection, FQs resistance testing and PMDT coverage in China, 2015–2019

Year	RR-TB cases detected			RR screening rate (%)			Proportion of FQs screening in RR-TB cases (%)			Prefecture-level coverage rate for PMDT (%)
	All	New cases *n* (%)	High-risk groups *n* (%)	All	New cases	High-risk groups	All	New cases	High-risk groups	
2015	10 019	3971 (39.6)	6048 (60.4)	29.5	23.1	57.3	57.8	58.6	57.2	70.0
2016	11 423	4564 (40.0)	6859 (60.0)	28.6	22.8	54.8	59.6	60.3	59.0	70.0
2017	13 069	6227 (47.6)	6842 (52.4)	37.8	33.1	57.2	51.9	52.7	51.1	78.0
2018	15 066	7807 (51.8)	7259 (48.2)	62.8	60.2	72.6	48.2	51.3	45.0	90.0
2019	18 623	10 204 (54.8)	8419 (45.2)	81.9	80.4	88.4	41.0	42.7	39.0	95.0
Total	68 200	32 773 (48.1)	35 427 (51.9)	49.4	45.3	67.2	50.3	51.0	49.6	–

*RR-TB* Rifampicin-resistant tuberculosis, *FQs* Fluoroquinolones, *PMDT* Programmatic Management of Drug-resistant Tuberculosis

### Comparison with the number of RR-TB patients estimated by the WHO

**Fig. 1:**
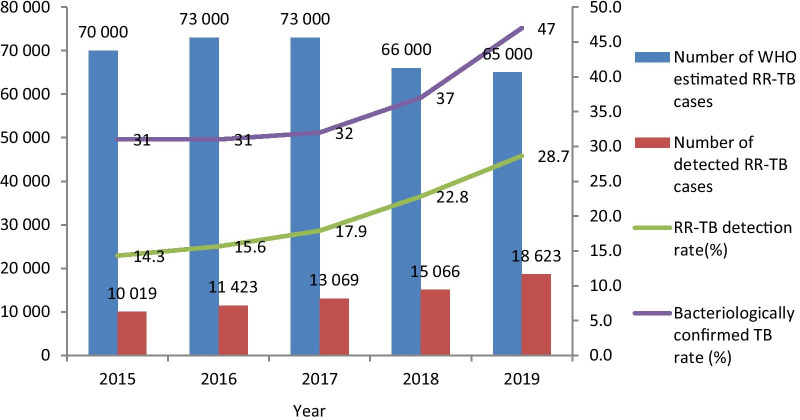
Comparison between the number of RR-TB cases detected and the WHO estimated, 2015–2019. Number of WHO estimated RR-TB cases, bacteriologically confirmed TB rate: from annual WHO TB report. *RR-TB* Rifampicin resistance tuberculosis

The rate of bacteriologically confirmed TB cases increased significantly from 31% in 2015 to 47% in 2019. At the same time, the number of RR-TB cases increased year by year. Comparing the number of detected RR-TB cases with the WHO estimate, it can be found that the gap narrowed year by year. The annual detection rates of RR-TB cases were 14.3%, 15.6%, 17.8%, 22.8% and 28.7% respectively (Fig. 1).

### DST methods and time to RR results notification

From 2015 to 2019, 52.2% of all patients used traditional phenotypic DST, which was slightly higher than that of rapid DST (47.8%). Although traditional DST was the main method, the application of rapid DST increased year by year, from 24.0% in 2015 to 67.1% in 2019, and the proportion of rapid DST has exceeded that of traditional DST since 2018. In 2019, the proportion of RR detected by rapid DST was 67.1%, which was 2.0 times that of the traditional method (67.1%/32.9%). The changes in the proportion of applying rapid DST annually were significant (Chi-square trend test, *P* < 0.0001) (Table 2).

**Table 2:** DST method and the source of presumptive RR-TB cases

	2015	2016	2017	2018	2019	Total	*P* value
	(*n* = 10 019)	(*n* = 11 423)	(*n* = 13 069)	(*n* = 15 066)	(*n* = 18 623)	(*n* = 68 200)	
DST method							
Rapid	2407 (24.0)	3714 (32.5)	5623 (43.0)	8378 (55.6)	12 501 (67.1)	32 623 (47.8)	0.000
Traditional	7612 (76.0)	7709 (67.5)	7445 (57.0)	6688 (44.4)	6122 (32.9)	35 577 (52.2)	
The source of presumptive RR-TB cases							
County-level hospital	6224 (62.1)	6722 (58.8)	7469 (57.2)	11 133 (73.9)	14 155 (76.0)	45 703 (67.0)	0.000
Prefecture-level and above hospitals	3795 (37.9)	4701 (41.2)	5600 (42.8)	3933 (26.1)	4468 (24.0)	22 497 (33.0)	

Data are presented as *n* (%)

*DST* Drug susceptibility testing, *RR-TB* Rifampicin-resistant tuberculosis

**Fig. 2:**
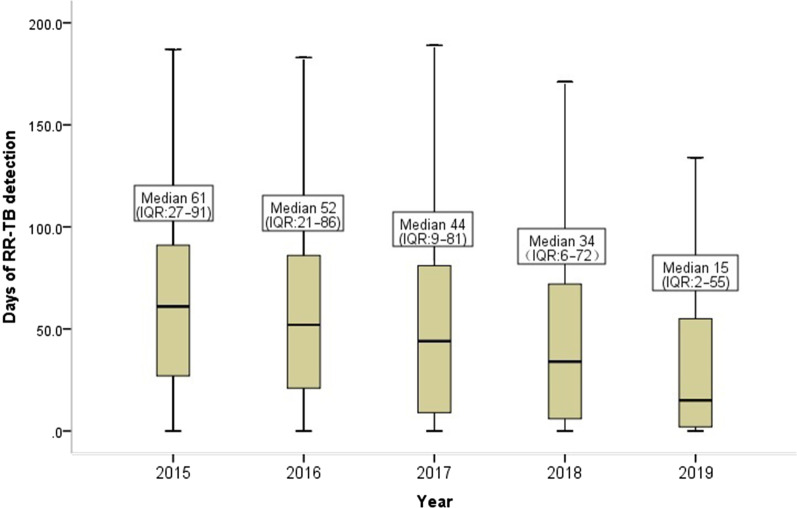
Days of RR-TB diagnosis: the time interval from the reporting time of sputum smear results to the reporting time of rifampicin susceptibility results. *RR-TB* Rifampicin resistance tuberculosis

The time interval from the sputum smear results notification to the obtaining of rifampin susceptibility results reduced year by year. From 2015 to 2019, the median time was 61 days (IQR: 27–91), 52 days (IQR: 21–86), 44 days (IQR: 9–81), 34 days (IQR: 6–72), and 15 days (IQR: 2–55) respectively. There was a significant difference in the time of obtaining DST results in each year (Kruskal Wallis test,* P* < 0.0001) (Fig. 2).

### Change in the source of presumptive RR-TB cases

In 2015–2019, the source of presumptive RR-TB cases showed different changes at different stages. During 2015–2017, the proportion of RR-TB cases diagnosed as presumptive RR-TB in county-level TB designated hospitals decreased slightly, from 62.1% in 2015 to 57.2% in 2017. But from 2018, this trend has reversed and showed a sharp rise, reaching a maximum of 76% in 2019. The change in the source of presumptive RR-TB annually was significant (Chi-squared test, *P* < 0.0001) (Table 2).

## Discussion

This study analyzed the characteristics of RR-TB case detection at the level of PMDT after 2015, especially after implementation of the national 13th Five-Year TB Program in 2016 in China.

Our analysis indicated that the RR screening rate and the number of RR-TB cases detection have increased significantly from 2015 to 2020. In 2019, the screening rate of RR in all bacteriologically confirmed TB cases reached 81.9%, exceeding the global level of 61% [2], and has become a routine work of the National TB Program (NTP). Delay in the diagnosis of RR-TB is a significant cause for the death, loss, and inappropriate treatment for RR-TB cases [22–24], as well as increases in the risk of drug resistance spreading in the community. In 2019 the median time for RR-TB cases to obtain RR results have been significantly reduced to 15 days, which is lower than the 26 days in the Republic of Korea [25]. In Shanghai, China, a metropolis with a population of 30 million, the diagnosis time has been as short as 9 days [26].

The main reason for the above changes is that after the national drug resistant cases detection policy was modified, in order to achieve the national 13th Five-Year TB Program targets the governments at all levels strengthened their commitment, especially near the end of the 13th Five-Year TB Program. This was done by increasing the capabilities of laboratories in weak areas and the aggressive scale up of the use of rapid molecular DST tools. From 2015 to 2018, the Chinese government has invested a total of about 260 million CNY to support economically underdeveloped areas such as the central and western regions to equip rapid molecular diagnostic tools [27]. Furthermore, China’s central government provided funds for the transportation costs of the county-level sputum specimens and strains and raised funds from various sources to provide free screening for presumptive RR-TB cases. These comprehensive measures improved the detection of RR-TB. Although the RR screening rate among high-risk groups has not yet reached the target of the 13th Five-Year TB Program, it will be achievable given the right strategic focus complemented by sustained leadership and adequate resources in 2020.

Our analysis also revealed that the source of presumptive RR-TB cases has changed in 2015–2019. More and more presumptive RR-TB cases are diagnosed in county-level hospitals, and the proportion is as high as 76.0% in 2019. The main reason for this trend is that the 13th Five-Year TB Program called for improving TB graded diagnosis and treatment services in order to strengthen the integration of TB prevention and treatment and make more rational use of medical resources [10]. By enhancing the capacity of county-level TB designated hospitals and the differentiation of medical insurance reimbursement to guide patients visiting hospitals in their jurisdiction to improve the accessibility of health care. This finding is of significance to China’s future deployment of PMDT. Although the diagnosis of drug-resistant TB is set at the prefecture level, it is necessary to include county-level rapid rifampicin susceptibility testing so that RR cases can receive appropriate treatment early and thus reduce the spread of drug resistance. Only by improving the TB diagnosis capabilities of county-level hospitals “missing RR-TB cases” can be reduced.

Our analysis also found some concerning issues. First, the proportion of new RR-TB cases has reached 54.8% in 2019. New RR-TB cases represent primary rifampicin resistance, suggesting that we need to pay attention to the spread of drug-resistant tuberculosis. Some studies in China have shown the transmission of multidrug-resistant tuberculosis [28, 29]. Our analysis results also indicate that the potential risk of community transmission of RR-TB in China is relatively high in recent years. China currently has no legislation on isolation treatment or travel restrictions for infectious RR-TB cases. It is imperative that China explores the possibility of legislation to manage infectious RR-TB in the future.

A second key concern is the continued low detection rate of RR-TB cases. 2019 was the year with the largest number of cases diagnosed, but only 28% of the RR-TB patients estimated by WHO were detected. This suggests that more than 70% of RR-TB cases were being missed. The main reasons are, first, that the rate of bacteriologically confirmed TB cases in China was low. It was 47% in 2019, which is lower than the global rate of 57% [2]. Second, 5% of prefectures nationwide cannot carry out DST, and 17% of prefectures have just gained the ability for DST in 2018–2019. The likelihood of insufficient personnel capacity and experience will affect the patient’s diagnosis. Third, there are limitations on current drug-resistance diagnostic algorithms regarding utilization of Xpert. Xpert is just a tool for the diagnosis of RR in bacteriologically confirmed TB in China and isn’t used in the diagnosis of signs or symptoms of TB as recommended by the WHO to maximize the detection of TB and RR-TB cases [16]. Xpert is still an expensive tool for China because the number of people with presumptive TB symptoms in China is enormous (about 3 million people per year).

A third issue relates to the FQs susceptibility testing. Contrary to the increasing RR screening rate, the FQs testing proportion has been declining year by year from 2016 and only 41.0% of RR-TB cases were tested for FQs susceptibility in 2019. This was far lower than the 71% globally [2], and was lower than the 44% in South Africa [30]. According to WHO’s good susceptibility testing coverage standards, although China’s RR screening rate has exceeded 80%, due to the low FQs susceptibility testing rate, China has not yet reached good testing coverage [2].

The reason why the ratio of rifampicin and FQs drug susceptibility testing showed opposite trends may be related to the fact that rifampicin testing increasingly uses molecular testing tools and can be conveniently implemented in county-level hospitals, hence its increased usage. FQs drug susceptibility testing is carried out at the prefecture level, and because traditional drug susceptibility methods can simultaneously obtain drug susceptibility results of other drugs, FQs drug resistance testing in China currently relies more on traditional methods. These factors may have contributed to the difference in the ratio of resistance testing of the two drugs. Another reason for the low proportion of FQs susceptibility testing is that the FQs screening rate is not a target indicator for the 13th Five-Year TB Program. Despite testing of FQs susceptibility, the result was not registered in the TBIMS in time. In addition, similar to the reason for the lower RR-TB detection, the DST capability of some newly implemented PMDT prefectures, especially second-line drug susceptibility testing, still need to be improved. In addition to the above reasons, since FQs susceptibility testing is currently not free, this also leads to a lower proportion of FQs testing.

FQs are the backbone of RR treatment regimens and FQs resistance is associated with poor treatment outcomes. WHO recommended that, before initiating treatment for RR-TB cases, it is necessary to carry out susceptibility testing for FQs, preferably by rapid assay [31]. The resistance rate of FQs among RR-TB patients in China is 27.4% [32], which is higher than the global average of 21% [2]. This implies that at least 1/4 of RR-TB cases enrolled for treatment that have not undergone FQs susceptibility testing will have a risk of poor treatment outcomes. China has introduced the short-course 9–12 month chemotherapy regimen recommended by the WHO. The key eligible criterion for using the short-term regimen is that the patient is not resistant to FQs. Therefore, in order to enable patients to apply appropriate chemotherapy regimens to ensure a therapeutic effect, it is imperative to conduct FQs susceptibility testing for RR-TB patients.

There are some limitations in our study. We used data from the TBIMS. The registration system only classifies the RR testing as “fast” and “traditional”, and it is not yet possible to distinguish whether the FQs susceptibility testing is “molecular” or “traditional”. If we need to understand the current ability of FQs rapid molecular DST in China, a special investigation needs to be conducted. In addition, the current system is unable to obtain data about whether the results of RR are directly diagnosed at the county level or by transporting specimens to prefectures for diagnosis. This data is meaningful for the national budget for the delivery of sputum specimens and the layout of rifampicin resistance testing institutions.

## Conclusions

Our analysis demonstrated that China’s political commitment together with aggressive 13th Five-Year TB Program targets, have had a great impact on the detection of RR-TB cases in China. After the change in RR-TB detection strategy, it exhibited a significant increase in the screening rate of RR and the number of RR-TB cases. At present, screening of RR is routine work for NTP. Nonetheless, there is still a gap in the detection of RR-TB cases. FQs susceptibility testing is another key concern in China’s current DST. In order to achieve the goal of END TB, China also needs to reduce the spread of RR-TB and effectively strengthen the laboratory capability at the county-level to allocate health resources reasonably.

## Data Availability

All data during this study are included in this published article.

## Abbreviations

RR-TB: Rifampicin resistance tuberculosis
PMDT: Programmatic Management of Drug-resistant Tuberculosis
DST: Drug susceptibility testing
TBIMS: Tuberculosis Information Management System
FQs: Fluoroquinolones
TB: Tuberculosis
WHO: World Health Organization
13th Five-Year TB Program: 13th Five-Year National Tuberculosis Prevention and Control Program (2016–2020)
Xpert: Xpert MTB/RIF
LPAs: Line probe assays
MeltPro: MeltPro TB assay
NTP: National TB Program
NTM: Non-tuberculous mycobacteria
